# In vivo phosphoproteome characterization reveals key starch granule-binding phosphoproteins involved in wheat water-deficit response

**DOI:** 10.1186/s12870-017-1118-z

**Published:** 2017-10-23

**Authors:** Guan-Xing Chen, Shou-Min Zhen, Yan-Lin Liu, Xing Yan, Ming Zhang, Yue-Ming Yan

**Affiliations:** 1 College of Life Science, Capital Normal University, Xisanhuan Beilu No. 105, 100048 Beijing, People’s Republic of China; 2 Hubei Collaborative Innovation Center for Grain Industry/Yangtze University, Jingzhou, 434025 China

**Keywords:** *Triticum aestivum* L., Water-deficit, Starch granule-binding proteins, Phosphoproteome, Bioinformatics

## Abstract

**Background:**

Drought stress during grain development causes significant yield loss in cereal production. The phosphorylated modification of starch granule-binding proteins (SGBPs) is an important mechanism regulating wheat starch biosynthesis. In this study, we performed the first proteomics and phosphoproteomics analyses of SGBPs in elite Chinese bread wheat (*Triticum aestivum* L.) cultivar Jingdong 17 under well-watered and water-stress conditions.

**Results:**

Water stress treatment caused significant reductions in spike grain numbers and weight, total starch and amylopectin content, and grain yield. Two-dimensional gel electrophoresis revealed that the quantity of SGBPs was reduced significantly by water-deficit treatment. Phosphoproteome characterization of SGBPs under water-deficit treatment demonstrated a reduced level of phosphorylation of main starch synthesis enzymes, particularly for granule-bound starch synthase (GBSS I), starch synthase II-a (SS II-a), and starch synthase III (SS III). Specifically, the Ser34 site of the GBSSI protein, the Tyr358 site of SS II-a, and the Ser837 site of SS III-a exhibited significant less phosphorylation under water-deficit treatment than well-watered treatment. Furthermore, the expression levels of several key genes related with starch biosynthesis detected by qRT-PCR were decreased significantly at 15 days post-anthesis under water-deficit treatment. Immunolocalization showed a clear movement of GBSS I from the periphery to the interior of starch granules during grain development, under both water-deficit and well-watered conditions.

**Conclusions:**

Our results demonstrated that the reduction in gene expression or transcription level, protein expression and phosphorylation levels of starch biosynthesis related enzymes under water-deficit conditions is responsible for the significant decrease in total starch content and grain yield.

**Electronic supplementary material:**

The online version of this article (10.1186/s12870-017-1118-z) contains supplementary material, which is available to authorized users.

## Background

Wheat (*Triticum aestivum* L.) grain yield and quality are affected by many environmental factors. Among the external stresses, water-deficit is one of the most important stress factor, as it causes significant yield loss in cereal production [1, 2]. The reductions of grain yield are due mainly to reduced starch accumulation, with starch representing 65–75% of grain dry weight [3, 4]. Thus, water stress affects starch biosynthesis and the composition of grain starch [1, 5].

Starch consists of the glucose polymers amylose and amylopectin. Amylose, as a relatively linear molecule composing of (1–4)-linked units of d-glucopyranosyl, is produced by GBSS I [6]. Amylopectin consists mainly of long chains of (1–4)-linked d-glucopyranosyl units with occasional branching (1–6) linkages, resulting in tandem linked clusters (~9–10 nm long each) [7, 8]. Four key enzymes, ADP-glucose pyrophosphorylase (AGPase), SS, starch-branching enzyme (SBE), and starch-debranching enzyme (DBE) are involved in amylopectin synthesis [9].

Based on its diameter, starch granules can be classified into three types: A-granules with 10–50 μm in diameter representing up to 70% of the volume and 10% of the total number of starch granules [10, 11]; B-granules with diameter 5–9 μm and accounting for about 30% of the volume and 90% of the total number of granules, and C-granules with less than 5 μm in diameter. In general, C-granules are difficult to isolate and quantify, leading to misclassification as B-granules [12, 13]. For B-granules, the amylose content is 5% less than in A-granules [14].

Protein phosphorylation is one of the most common post-translational modifications (PTMs) in vivo, which regulates different biological processes, such as transcription and translation, proliferation, cellular signaling, communication and differentiation [15]. Phosphorylation modifications in eukaryotes mostly occur at the residues of serine (Ser), threonine (Thr), and tyrosine (Tyr).

To date, the phosphoproteins in starch are detected mainly by three methods: Pro-Q Diamond staining, phosphorylation isotope labeling by γ-^32^P-ATP in vitro, and liquid chromatography-mass spectrometry (LC-MS/MS) technology [16–19]. For example, GBSS I can be stained with a phosphoprotein-specific dye in maize (*Zea mays* L.) [17]. These phosphorylated enzymes (proteins) binding starch granules labeled by γ-^32^P-ATP in vitro can improve amylase activity and amylose synthesis [16, 20, 21].

In a recent study in our laboratory, we identified phosphorylation sites of GBSS I and SS I by LC-MS/MS which play important roles in starch biosynthesis [18, 22]. In bread wheat, large-scale phosphoproteomic analyses of seeds and seedling leaves have been performed using phosphopeptide enrichment and LS-MS/MS [15, 23–27]. More recently, we performed the first comparative proteomics analysis of A- and B-type starch granule-associated proteins in wheat and *Aegilops crassa* through starch granule-binding protein (SGBP) extraction and two-dimensional gel electrophoresis (2-DE) maps [19]. However, because the enrichment of starch granule-binding proteins with high purity in sufficient quantity from starch granules is difficult, characterization of the phosphoproteome of SGBPs in vivo during wheat grain development and under water-deficit has been limited.

In the present study, we reported the first phosphoproteome analysis of SGBPs in vivo in developing wheat grains subjected to water-deficit treatment. We determined the dynamic transcriptional expression profiles of the key genes associated with starch biosynthesis during grain development and under water-deficit treatment. Our results revealed the changes of SGBP phosphorylation level in vivo in response to drought stress, as well as starch biosynthesis, which provides new insights into the molecular mechanisms of the response to drought stress.

## Methods

### Plant materials, planting, and sampling

The elite Chinese winter wheat cultivar Jingdong 17 with good drought resistance was used as material and planted in winter at the experimental station of China Agricultural University during the 2014–2015 growing season from October to June of next year, with three biological replicates (each plot was 30 m^2^). The soil is glue test bed for low light loamy salinization soil, and the cultivation and management practices used were the same as the local wheat management practices. Two treatments were imposed at the jointing and flowering stages: well-watered with regular water irrigation (CK) and water-deficit without irrigation (DS). The average annual amount of sunshine at the experimental location is 2690 h, the average annual temperature is 12.5 °C, and average rainfall during the wheat growing season is approximately 150 mm. To harvest grain materials at different developmental stages, individual flowers were marked with colored tape at post-anthesis. Developing grains from the middle of spikes were harvested at 8, 10, 15, 20, 25, and 30 days post-anthesis (DPA) from 9:00–11:00 am, and stored at −80°C.

### Measurement of soil water content, agronomic traits, and quality

The measurement of soil water content during different development stages, including the double-ridge, jointing, flowering, and maturity stages, followed the previously described soil drilling method of Guan et al. [28].

The physiological traits of flag leaves and relative water content at five time points after flowering under water-deficit and well-watered conditions were measured according to Larbi and Mekliche [29]. The main phenotypic and agronomic traits of mature plants were recorded, including the number of spikelets, infertile spikelets, and kernels per spike. We also recorded data on grain weight per spike, weight of 1000 kernels, and grain yield. Statistical analyses were conducted with SPSS statistics software (version 17.0). Total starch and amylose content were measured with assay kits (Megazyme Int. Ireland, Ireland), according to the manufacturer’s protocol and Chen et al. [22]. Farinograph parameters were obtained by analyzing 10-g samples using a Farinograph-E instrument (C.W. Brabender Instruments, Inc.), based on the American Association of Cereal Chemists Approved Method (2000) 54 21.

### Purification of starch granules, ultrastructural observation by SEM, and extraction of SGBPs

Starch granules from three biological replicates at various grain development stages were separated and purified according to Chen et al. [18]. Scanning electron microscopy (SEM) examination of starch granules was performed according to Chen et al. [22] with a Hitachi Model S-4700 scanning electron microscope. SGBPs were extracted and purified according to the previous reports*.* [19, 22, 30].

### 2-DE and protein identification by MALDI-TOF/TOF-MS

Two-dimensional gel electrophoresis (2-DE), image analysis, and matrix-assisted laser desorption/ionization time-of-flight/time-of-flight mass spectrometry (MALDI-TOF/TOF-MS) identification of extracted SGBPs in three biological replicates were performed according to Hao et al. [31]. For the linear gradient IPG strip, each sample includes 50 μg SGPBs in 360 μL rehydration buffer. For each biological sample, the 2-DE experiments were repeated three times for error control. The gels were stained with CBB (R-250/G-250 = 4:1) and analyzed the expression quantity using ImageMaster 2D Platinum software version 7.0 (Amersham Biosciences).

The differentially expressed protein spots were excised and analyzed according to the recent reports [22, 30]. Statistical significance was determined according to the Protein Score C.I. % and Total Ion Score C.I. %, both above 95% and a significance threshold of *p* < 0.05 for the MS/MS [32].

### Phosphopeptide enrichment using TiO_2_ microcolumns

The enrichment procedure for phosphopeptides from three biological replicates was performed as reported by Wu et al. [33] and Zhang et al. [26]. SGBPs extracted at 20 DPA were reduced directly with dithiothreitol (DTT), alkylated with iodoacetamide, and, subsequently, digested with endoproteinase Lys-C and trypsin. TiO_2_ beads (GL Sciences, Tokyo, Japan) were incubated in 400 μL of loading buffer, containing 65% acetonitrile (ACN) and 2% trifluoroacetic acid (TFA), saturated with glutamic acid. A total of 3 mg of tryptic peptides from each biological replicate was dissolved in 600 μL of loading buffer, and then incubated with the appropriate amount of TiO_2_ beads. After washing with 600 μL of wash buffer (65% ACN, 0.1% TFA), the phosphopeptides were eluted twice with 300 μL of elution buffer (500 mM NH_4_OH, 60% ACN). The eluates were dried and reconstituted in 0.1% formic acid (FA) in H_2_O for MS analysis.

### Phosphopeptide identification, phosphorylation site localization, and Bioinformatic analysis

The enriched phosphopeptides of each biological replicates were separated on a self-packed C18 reversed-phase column that was directly connected with a nanoelectrospray ion source in an LTQ-Orbitrap XL mass spectrometer (Thermo Fisher Scientific, America). The inner diameter of reversed-phase column is 75 μm and 150 mm length (Column Technology Innovation CTI, Fremont, CA). The pump flow was split to achieve a flow rate of 1 μL/min for sample loading and 300 nL/min for MS analysis. The mobile phases consisted of A (0.1% FA) and B (0.1% FA/80% ACN). A five-step linear gradient was applied (from 5% to 30% B in 105 min, 35% to 90% B in 16 min, 90% B in 4 min, 90% to 2% B for 0.5 min, and 2% B for 14.5 min). The spray voltage was set to 2.0 kV, and the temperature of the heated capillary was set to 240 °C. Every MS scan was acquired at a resolution of 60,000 (at 400 m/z) with the lock-mass option enabled, followed by top 10 data-dependent MS/MS scans performed using collision induced dissociation (CID). We set 500 as the threshold for precursor ion selection, and the mass window for precursor ion selection was 2.0 Da. The dynamic exclusion duration was 120 s, the repeat count and duration was 1 and 30s, respectively. The analyzer for the MS scans was Orbitrap, and the analyzer for the MS/MS scans was LTQ (37% relative collision energy). Three biological replicates were performed independently from sample collection to phosphopeptide identification. Raw files were processed using MaxQuant (version 1.2.2.5) software, according to Cox et al. [34], and then searched against the wheat database (77,037 entries).

Phosphorylation residue localization was evaluated based upon the post-translational modification (PTM) scores. Potential phosphorylation residues were then grouped into three categories based upon Schwartz et al. [35] and Sugiyama et al. [36].

Phosphopeptides that met the following conditions were considered as having undergone a significant change in phosphorylation level, based on the method of Lv et al. [23]: (1) phosphopeptide detected in all three biological replicates; (2) phosphopeptides with credible Student’s *t*-test (*p* < 0.05), (3) phosphorylation localization probability ≥0.75, and (4) phosphorylation site score differences ≥5.

The motif-X algorithm (http://motif-x.med.harvard.edu/) was used to extract significant enriched phosphorylation motif from phosphopeptides [37]. A Phyre2 online server was used to predict the 3D structures of the proteins [38] and the 3D structures and the phosphorylated sites were displayed using Swiss-PdbViewer (SPDBV version 4.1) software [39].

### Antibody development, western blotting, and immunolabeling

Polyclonal antibodies were produced in rabbits derived from the N-terminal sequences of *T. aestivum* SBE I, and *T. aestivum* SBE IIa [40]. Monoclonal antibody anti-wheat GBSS I, SS I, and SS II antisera were prepared as described by Li et al. [41]. After staining, the expected protein bands were collected and digested with trypsin, and then identified by MALDI-TOF/TOF-MS as described above. The identified proteins were further confirmed by Western blotting according to Li et al. [41].

Immunolabeling experiments were performed with samples of 15 DPA grains, using the recently described method by Chen et al. [18]. The primary antibodies of GBSS I were diluted to 1:3000. In the control experiment, the primary antibody was omitted to test for nonspecific secondary antibody binding [42, 43].

### RNA extraction, cDNA synthesis, and real-time quantitative reverse transcription polymerase chain reaction (qRT-PCR)

Developing grains from the central parts of the spikes were harvested at six developmental stages of Jingdong 17 (8, 10, 15, 20, 25, and 30 DPA). Total RNA of individual samples was extracted with TRIzol Reagent (Invitrogen) according to Li et al. [41]. cDNA were synthesised based on Chen et al. [22]. Transcriptional expression patterns of the selected genes were detected using qRT-PCR according to Chen et al. [18] with minor modifications. The genes and the candidate reference genes were listed in Additional file 1: Table S1.

## Results

The strategy and experimental design of this study are presented in Additional file 2: Figure S1. Developing Jingdong 17 grains were harvested at 25 DPA from plants grown under water-deficit and well-watered conditions. Starch granules were then separated and purified. SGBPs were extracted and separated by 2-DE, and identified by MALDI-TOF/TOF-MS. Subcellular localization of key SGBPs was determined using immunogold labeling. Subsequently, SGBPs were subjected to large-scale phosphoproteome analysis with TiO_2_ microcolumns enrichment and LC-MS/MS, and we studied phosphorylated SGBPs that regulate starch biosynthesis and drought stress responses. We determined the dynamic transcriptional expression profiles of key genes related to starch biosynthesis during grain development under the two conditions.

### Dynamic changes in soil water and leaf water content

**Fig. 1:**
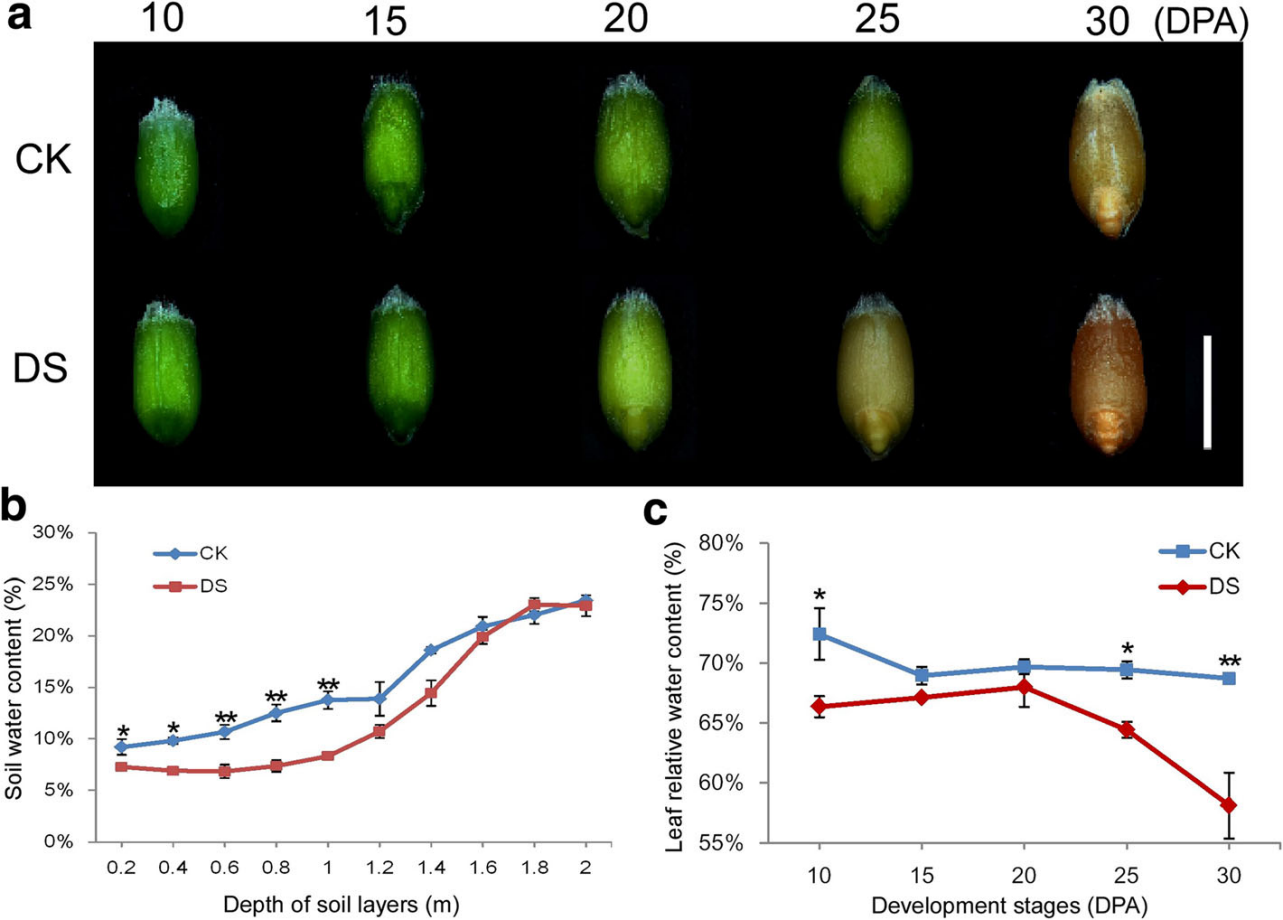
The morphological pictures of the development grains and comparative analysis of soil water content and relative water content of Jingdong 17 leaves under well-watered and water-deficit conditions. **a** The morphological pictures of development grains under well-watered and water-deficit conditions. **b** Comparative analysis of soil water content. **c** Comparative analysis of leaf relative water content

Grain developmental stages and soil water content are shown in Fig. 1. The seeds of plants subjected to water-deficit treatment ripened faster than the seeds under well-watered conditions (Fig. 1a). Soil water content increased gradually with depth of the soil layers, from 0 to 200 cm. In the 0–100 cm soil layer, the water content ranged from 7% to 8%, 9% to 14%, under water-deficit condition and well-watered condition, respectively. Soil water content was approximately 20%–30% lower under water-deficit than under well-watered condition as shown in Fig. 1b. Thus, according to Guttieri et al. [44], the effect of the water-deficit corresponded to medium-to-severe drought stress. The medium-to-severe drought stress resulted in significantly reduced relative water content of the flag leaves (Fig. 1c).

### Effects of water-deficit treatment on yield traits and starch composition

Changes in main agronomic traits and starch contents in response to control and water-deficit treatments are shown in Table 1. These results indicated that some important agronomic traits related to yield were affected significantly by water-deficit. In particular, the 1000-kernel weight and grain yield were reduced significantly under water-deficit condition. Total starch and amylopectin contents were also reduced significantly. These results indicated that the water-deficit treatment could cause significant yield losses and premature grain development, at the key jointing and flowering stages.

**Table 1:** Comparative analysis of yield characters in Jingdong 17 under well-watered (CK) and water-deficit (DS) treatments

Treatments	Spikelet number	Kernels number	Kernels per spike	grain weight per spike (g)	TKW (Thousand kernel weight) (g)	Yield (Kg/acre)	Total Starch (%)	Amylopectin (%)	Amylose (%)
CK	17. 75 ± 1.98	14.87 ± 0.98	31.33 ± 3.34	1.19 ± 0.01	52.5 ± 6.32	10,854	69.91 ± 5.35	54.38 ± 3.97	15.81 ± 1.38
DS	17. 53 ± 2.31	13.90 ± 1.11	28.70 ± 3.12	1.16 ± 0.01	48.5 ± 4.23*	7938**	63.22 ± 4.54*	45.39 ± 2.99*	17.83 ± 1.55

*P < 0.05, **P < 0.01

We determined changes in the main farinograph parameters in response to the control and water-deficit treatments, (Table 2). As shown in Table 2, important farinograph parameters, including peak viscosity, minimum viscosity, attenuation, final viscosity, and retrograde values, were affected significantly by water deficit. But the parameters of attenuation, peak time and pasting temperature did not change significantly under water deficit in this study. And our results indicated that drought stress affected starch quality.

**Table 2:** Comparison of farinograph parameters of starch between the control (CK) and drought stress (DS) treatments

Materials	Peak viscosity	Minimum viscosity	Attenuation	Final viscosity	Retrogradation value	Peak time	Pasting temperature
CK	2381 ± 5	1858 ± 11	523 ± 4	3248 ± 12.3	1390 ± 4.5	6.4 ± 0.12	63.6 ± 1.44
DS	2198 ± 7*	1733 ± 8*	465 ± 8.2	3020 ± 11*	1287 ± 8.1*	6.4 ± 0.23	64.4 ± 1.52

*P < 0.05

### Comparative proteome analysis of SGBPs under water-deficit conditions

Starch granules were separated and purified, and then observed using SEM. Microscope images showed that the granules had no protein or cell debris contamination (Additional file 2: Fig. S2). After removing the surface-associated proteins by washing three times, SDS-PAGE analysis further confirmed the complete removal of all contamination (Additional file 2: Fig. S3). Moreover, SDS extraction and sonication did not change the visual appearance of starch granules observed by SEM.

**Fig. 2:**
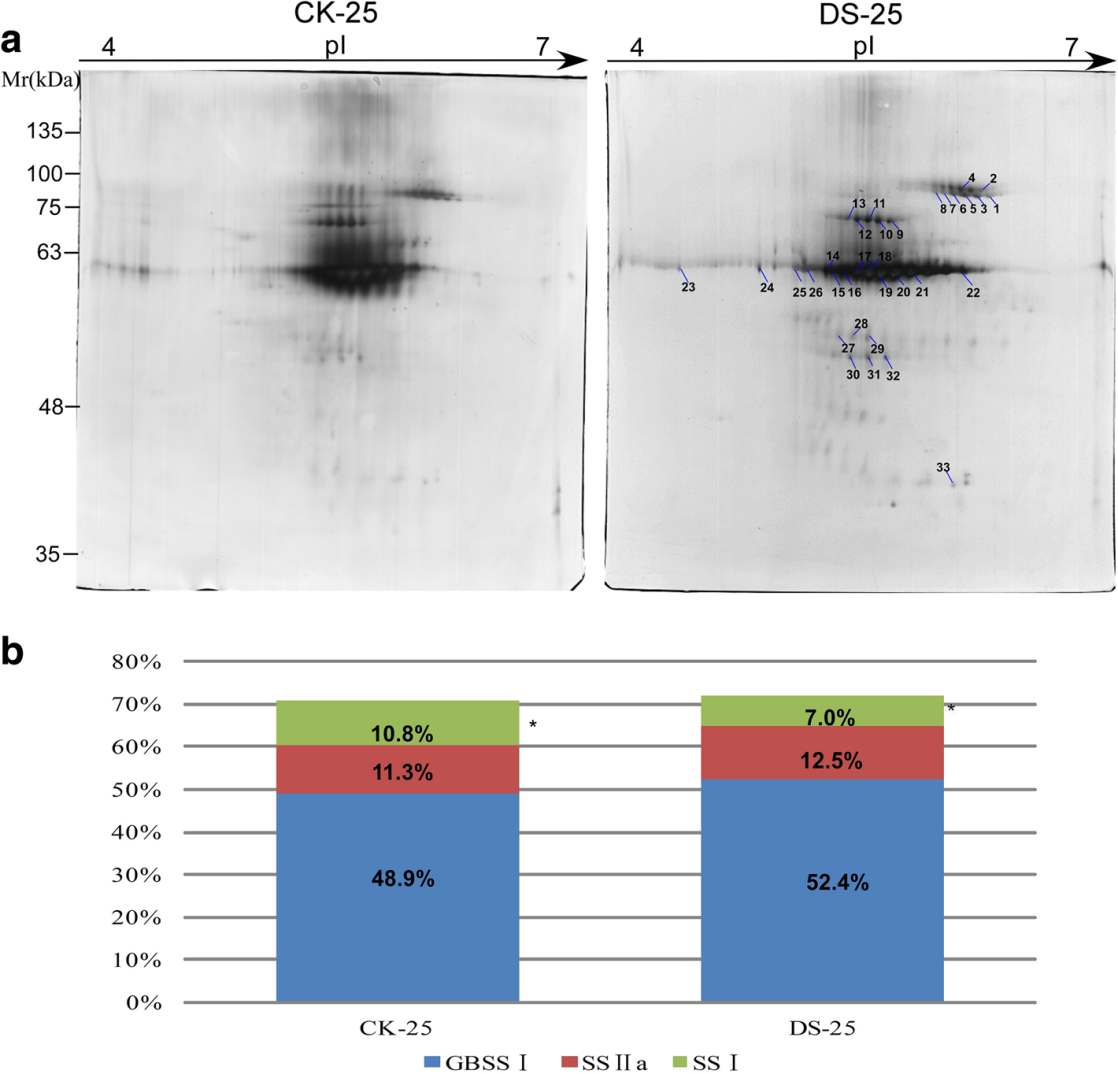
Comparison of the content of starch granule-binding protein under well-watered and water-deficit conditions. **a** 2-DE maps of the proteomes of starch granule-binding proteins under well-watered and water-deficit conditions. **b** Comparison of expression patterns of three types of starch granule-binding proteins, the asterisk showed the significant difference between the two groups

A 2-DE separation was performed using 60 μg of SGBPs extracted from developing grains at 25 DPA. The SGBPs displayed similar proteome profiles under the two treatments (Fig. 2a). In total, we identified 33 protein spots containing SS I, SS II-a, and GBSS I, of which 12 spots were identified as GBSS I. A total of five and eight spots were identified as SS I and SS II-a, respectively. The remaining spots were identified as GBSS I partial (Additional file 1: Tables S2 and S3). Further analysis showed that the GBSS I, SS I, and SS II-a contents accounted for 71 and 71.9% of total SGBPs for the well-watered and water-deficit treatments, respectively. However, the respective proportions showed greater differences. For the well-watered treatment, the waxy protein (GBSS I) accounted for approximately 48.9% of the total SGBP content, while, under drought stress, it increased slightly to 52.4%. SS II-a accounted for 11.3% under the well-watered treatment, and its content increased to 12.5% in response to water-deficit. SS I accounted for 10.8% in well-watered plots, but it decreased significantly to 7.0% for the water-deficit treatment (Fig. 2b).

### Phosphoproteome characterization of SGBPs under well-watered and water-deficit conditions

**Fig. 3:**
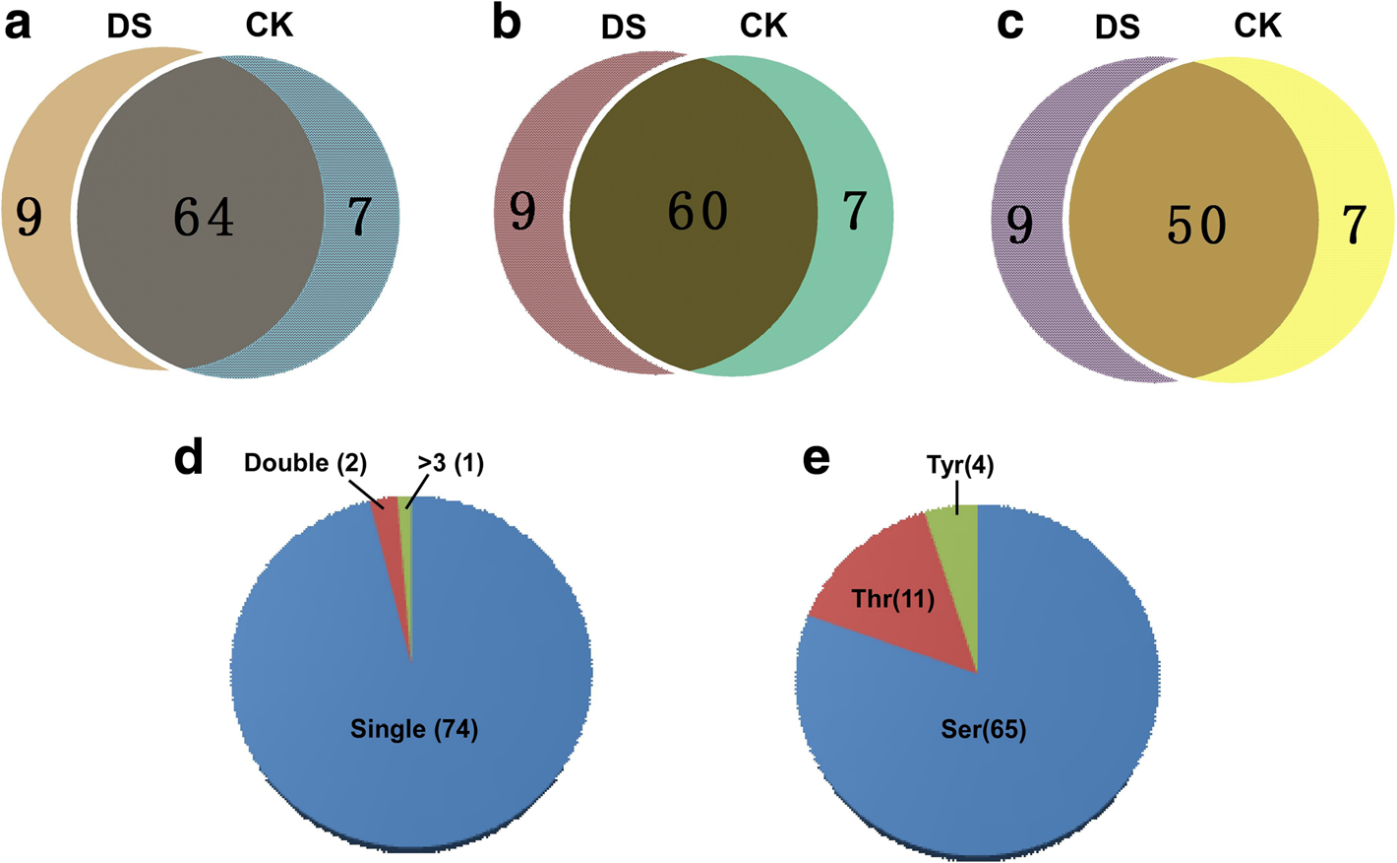
Phosphorylation analysis of starch granule-binding proteins under well-watered and water-deficit conditions. **a** Amino acids sequence with marked phosphorylated sites. **b** Phosphorylated peptide analysis of starch granule-binding proteins under well-watered and water-deficit conditions. **c** Phosphorylation site number analysis of starch granule-binding proteins under well-watered and water-deficit conditions. **d** Distribution of phosphorylation sites and numbers of serine, threonine, and tyrosine residues in the phosphorylated peptides. **e**. Distribution of phosphorylation sites and numbers of serine, threonine, and tyrosine residues

SGBPs (5 mg) were extracted from purified starch granules at 25 DPA (three biological replicates) and the phosphopeptides were enriched and analyzed by LC-MS/MS. In total, we identified 76 phosphopeptides containing 80 phosphorylated sites, representing 66 phosphoproteins (Fig. 3a–c). Under well-watered conditions, 67 phosphopeptides containing 71 phosphorylated sites and 57 phosphoproteins were identified (Additional file 1: Table S4). A total of 69 phosphopeptides with 73 phosphorylated sites were identified in 59 phosphoproteins under water-deficit conditions (Additional file 1: Table S4). Among the 76 phosphopeptides, 74 included one phosphorylated site, two had two phosphorylated sites, and one had more than three phosphorylated sites (Fig. 3d). In addition, among the 80 phosphorylated sites, 65 serines, 11 threonines, and 4 tyrosines were phosphorylated (Fig. 3e). To improve the accuracy of the dataset, only phosphorylation sites with *p* ≥ 0.75 were used in subsequent analyses. All mass spectrometry proteomics data obtained in this study (Additional file 1: Table S4) were deposited in the ProteomeXchange Consortium with the dataset identifier PXD002805 [39].

**Fig. 4:**
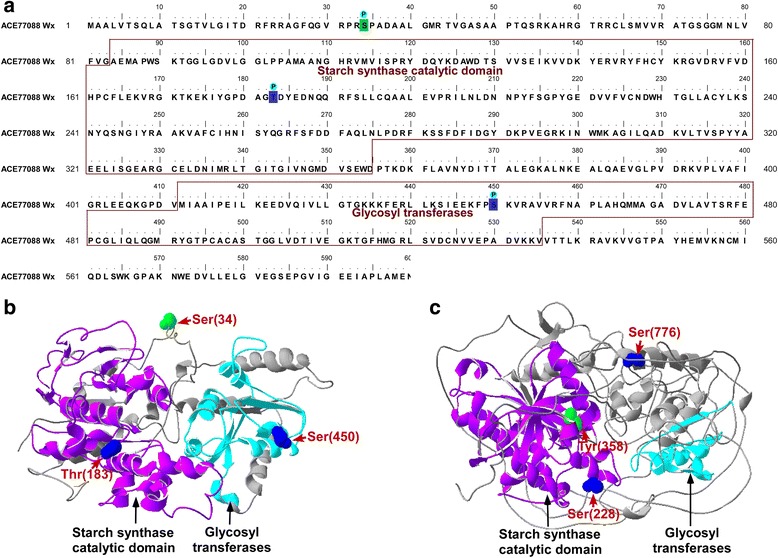
Phosphorylation of starch synthase-related enzymes. **a** Analysis of amino acid sequence of GBSS I (phosphorylated residues are marked). **b** 3D structure is shown for GBSSI. **c** 3D structure is shown for SS II-a

The identified phosphoproteins were mainly divided into seven categories: starch synthesis enzymes, protein kinase/phosphatase, ATPase, ubiquitin protein, transporter proteins, transcription/translation factors, and others (Additional file 1: Table S4). Starch synthesis enzymes included GBSS I, SS II-a, and SS III. The phosphorylation site distributions and 3-D structures of GBSS I and SS II-a are shown in Fig. 4. Three phosphorylated sites were detected under the water-deficit and well-watered conditions: Ser34, Ser450, and Thr323 in GBSS I (Fig. 4a, b) and Ser228, Ser776, and Tyr358 in SS II-a (Fig. 4c). It is worth noting that the degrees of phosphorylation at the Ser34 site in waxy protein, Tyr358 site in SS II-a, and the Ser837 site in SS III-a were reduced significantly in response to water-deficit conditions (Table 3). We also found that the degrees of phosphorylation of two kinase proteins (phosphatidylinositol 4-kinase type 2-beta and hydroxysteroid 11-beta-dehydrogenase) were reduced significantly in response to drought stress. The extent of phosphorylation at two sites in globulin 3 and one site in photosynthesis-related proteins PSII D2 increased significantly under the water-deficit treatment (Table 3).

**Table 3:** Comparative analysis of phosphorylated sites identified in Jingdong 17 under well-watered (CK) and water-deficit (DS) treatments

AC number	Protein descriptions	Modified sequence	DS/CK
CAT81057	Granule-bound starch synthase I	_S(ph)PADAPLGMR_	D
CAM32368	starch synthase IIa	_YGDYEEAY(ph)DVGVR_	D
EMS45039	Soluble starch synthase III	_LQSMLS(ph)LAR_	D
EMS49802	Nuclease domain-containing protein 1	_LSS(ph)FGLDR_	U
EMS55948	Serine/arginine-rich splicing factor 4	_RS(ph)ISPVAK_	U
EMS47146	Hydroxysteroid 11-beta-dehydrogenase 1-like	_WLTQTAS(ph)LR_	D
EMS51823	PSII D2 protein	_T(ph)IALGR_	U
EMS55513	Peroxisomal (S)-2-hydroxy-acid oxidase GLO1	_AIALTVDT(ph)PR_	U
ACJ65514	globulin 3	_PLLASLS(ph)KR_	U
		_S(ph)FHALAQHDVR_	U
EMS61682	putative calcium-binding protein CML7	_T(ph)PPSWVK_	U
EMT31973	Phosphatidylinositol 4-kinase type 2-beta	_NDS(ph)PLLLTK_	D
EMT25637	hypothetical protein	_APFGHS(ph)PVDPR_	U
EMS66158	hypothetical protein	_S(ph)HAAPTFTIK_	U
EMT16987	hypothetical protein	_VGLIRPNS(ph)PK_	D
EMT26644	hypothetical protein	_AIVQEEHVAS(ph)LPR_	U
EMT24427	hypothetical protein	_GVSYPY(ph)IMAWPR_	D

U: The spot is upregulated when DS compared with CK. D: The spot is downregulated when DS compared with CK

### Starch granule-binding proteins verification and western blotting

**Fig. 5:**
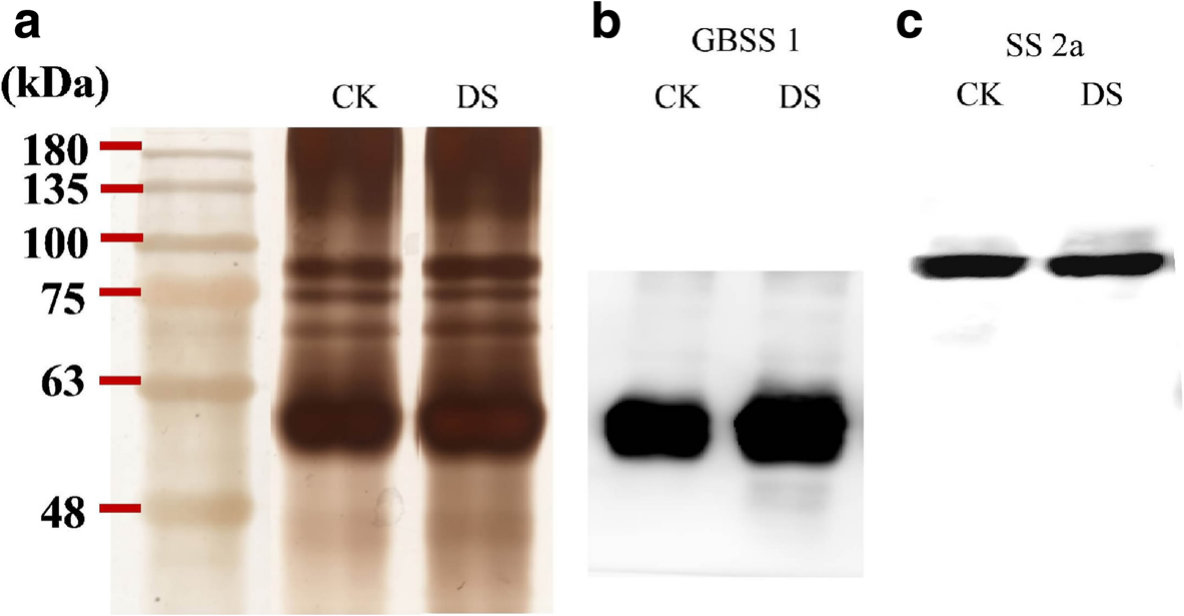
Starch granule-binding proteins from purified starch by electrophoretic analysis and western blot. **a** SDS-PAGE of starch granule-binding proteins under well-watered and water-deficit conditions. **b** Analysis of the protein level of GBSS I by western blotting. **c** Analysis of the protein level of SS II-a by western blotting

The SGBPs from 25 DPA were fractionated by SDS-PAGE and silver-stained (Fig. 5a). The isolated GBSS I and SS II-a proteins were identified by MALDI-TOF/TOF-MS (Additional file 1: Table S5). Moreover, monoclonal antibodies against GBSS I and SS II-a demonstrated their high specificity (Fig. 5b, c).

### Subcellular localization of GBSS I

**Fig. 6:**
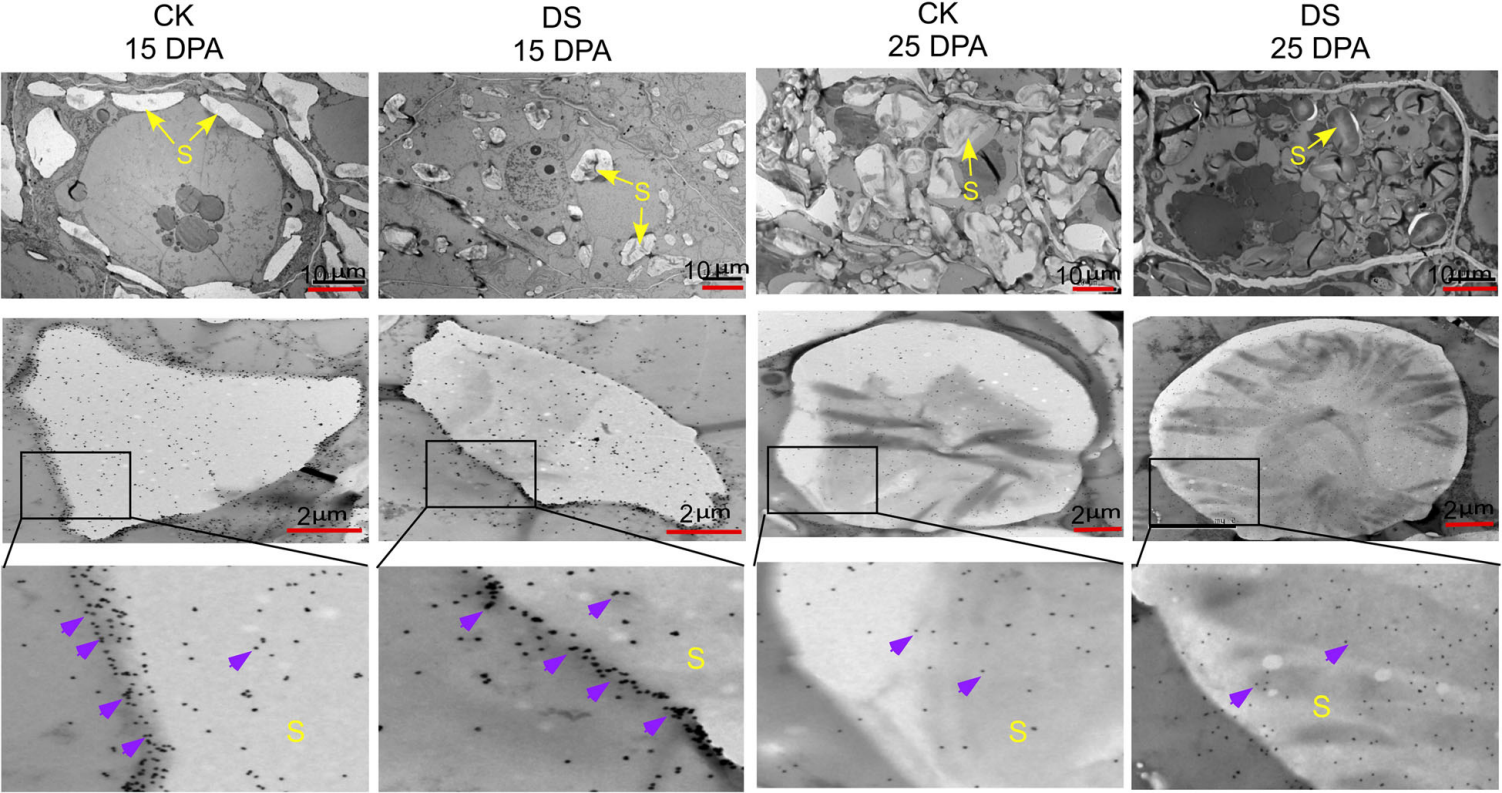
Immunolocalization of GBSSI in immature seeds (15 and 25 DPA). S, starch granules; PB, protein body. Triangular arrowheads indicate gold particles

The subcellular localization of GBSS I was determined by immunolocalization. As is shown in Fig. 6, the hybridization signal of GBSS I was present mainly around the starch granules, under both conditions at 15 DPA, while most signals were located within starch granules at 25 DPA (Fig. 6).

### Dynamic transcriptional expression profiling of starch synthesis-related genes during grain development

**Fig. 7:**
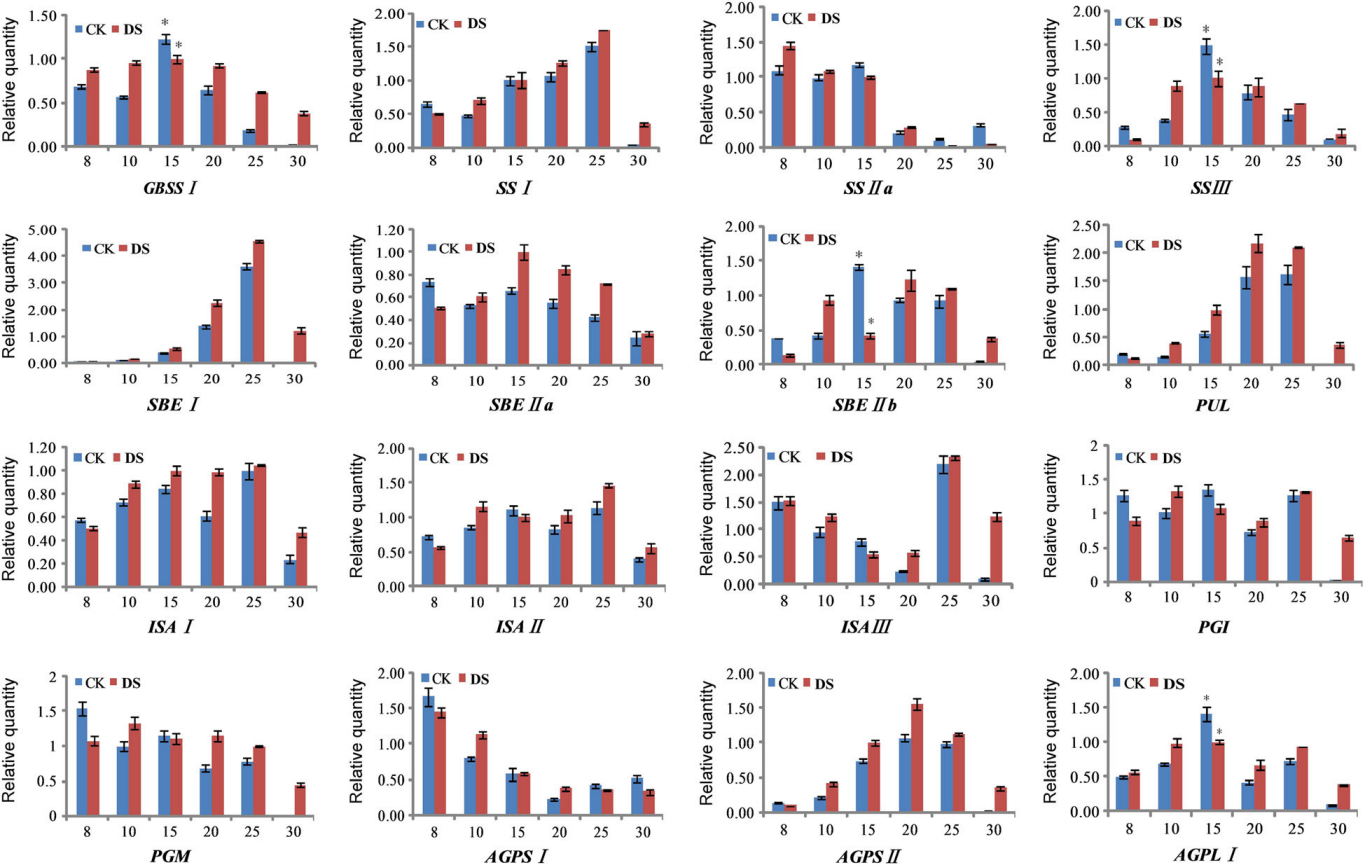
qRT-PCR analysis of genes related to starch synthesis in developing seeds from the Chinese wheat cultivar Jingdong 17. The star indicates significant difference between CK and DS groups

We determined the dynamic expression profiles of 16 starch synthesis-related genes, during six grain developmental stages in Jingdong 17 under both conditions by qRT-PCR analysis (Fig. 7). In general, all starch synthesis-related genes displayed a similar expression trend during grain development under both conditions. Most of the genes, such as *GBSS I*, *SS I*, *SS III*, *SBE I*, *SBE II-a*, *SBE II-b*, *Pullulanase* (*PUL*), *isoamylase I* (*ISA I*), isoamylase *II* (*ISA II*), *adenosine 5 diphosphate glucose pyrophosphorylase small subunit II* (*AGPS II*) and *adenosine 5 diphosphate glucose pyrophosphorylase large subunit I* (*AGPL I*), exhibited an up-downregulated expression trend and a higher expression level at the mid stages of grain development. And some genes (*SS II-a, PGM, AGPS I*) exhibited a downregulated expression trend. However, some genes exhibited expression differences between well-watered and water-deficit conditions. For example, *SS III*, *SBE II-b* and *AGPLI* genes were downregulated significantly at 15 DPA under water-deficit condition (Fig. 7).

## Discussion

Previous studies showed that drought stress severely affects wheat growth and development, especially at the grain filling stage, which generally results in a significant grain yield reduction. Photosynthesis is weakened in response to drought stress, and the accumulation of dry matter is reduced. Earlier studies showed that the grain filling rate and spike grain weight are reduced when drought stress is imposed at grain filling [45–47]. Continuous drought results in significant loss of total dry matter and wheat grain yield [48]. Similarly, our study showed that water-deficit contributed to a marked decline in wheat leaf water content, spike grain number, 1000-kernel weight, and grain yield (Table 1). Wheat endosperm consists of starch, which serves as a major contributor to grain yield. And our results showed that total starch and amylopectin contents were reduced significantly under water-deficit conditions (Table 1). And the previous studies had shown that drought stress could decrease the number of endosperm cells and influence the formation of amyloids at early stages of seed growth, which subsequently causes a decline in endosperm starch accumulation [49, 50]. The reduction in endosperm starch accumulation is the primary cause of the grain weight reduction under drought conditions [51]. Our study indicated that the significant reduction in amylopectins, which account for 70% of the starch content, could contribute to a marked reduction in total starch content and grain yield (Table 1).

As is known, starch is composed of amylopectins and amyloses, of which amylose synthesis is controlled by GBSS I, and amylopectins are synthesized by concerted reactions catalyzed by four enzyme classes: AGPase, SS, SBE, and DBE [52]. In this study, our proteomic results revealed that the amount of the protein SSI was compromised significantly under water-deficit conditions compared to well-watered conditions. While the amylose content increased sightly after water-deficit treatment. *GBSS I* expression was increased during all stages, with the exception of 15 DPA after drought stress treatment according to the results of qRT-PCR shown in Fig. 7. And the quantity of the GBSS I protein was also increased, but not significantly for the water-deficit treatment group (Fig. 2). Meanwhile, immunolocalization results suggest that GBSS I had a clear movement from the periphery to the interior of starch granules during grain development (Fig. 6).

The synthesis of amylopectins are mainly controlled and catalyzed by starch synthase (SS). And the SS group consists of four isoforms (SS-I, SS-II, SS-III, and SS-IV), which are localized at the granule surface predominantly [52–55]. In this study, we found that the content of SS I was reduced significantly in response to the water-deficit treatment (Fig. 2), which finally resulted in significant reduction of amylopectin and total starch content (Table 1). Studies involving *Arabidopsis thaliana* L. and *Oryza sativa* L. demonstrated that SSI is required for the elongation of short A-chains within amylopectin [56, 57]. The function of SS-II is elongation of amylopectin chains of DP 6–10 to produce intermediate-length chains of DP 12–25 [58]. Similar to the *GBSS I* gene, the transcriptional expression changes of several amylopectin synthesis-related genes such as *SS III*, *SBE II-b* and *AGPL I* were reduced significantly at 15 DPA (Fig. 7), suggesting that the effects of drought stress on starch biosynthesis occur at the transcriptional level, protein translation and posttranslational phosphorylation modification levels.

Phosphorylation could improve the activity of the SGBPs, thereby increasing the rate of starch synthesis and increasing total starch content [20, 21]. In a previous study, we used LC-MS/MS to identify phosphorylation sites present in GBSS I and SS I [18, 19]. In this study, we performed large-scale phosphoproteomic analysis of starch granule-binding proteins in Jingdong 17 in response to well-watered and water-deficit treatments. And our results indicated that the phosphorylation levels of key starch synthesis-related proteins were reduced significantly by the water-deficit treatment. Four types of enzyme related to starch synthesis, including GBSS I, SS II-a, SS III, and AGPS, were phosphorylated, and the phosphorylation sites were highly conserved [18, 19]. However, there were some differences in the degree of phosphorylation. For example, the Ser34 site of GBSSI, the Tyr358 site of SS II-a, and the Ser837 site of SS III-a had lower degrees of phosphorylation under drought conditions than under the well-watered treatment (Table 3). Based on previous study, the phosphorylation sites in starch synthase might play important roles in recognizing and attracting glucan substrates [18]. A study on the 14–3-3 protein showed that phosphorylation could increase the affinity between the interacting proteins [59]. For the GBSSI, reduced phosphorylation at Ser34 might affect enzyme activity, influence amylase synthesis, and reduce amylose content, for the reason that phosphorylation improves amylase activity and increases amylose synthesis [20]. Similarly, for amylopectin, reduced phosphorylation at Tyr358 of SS II-a could also affect enzyme activity, influence amylopectin synthesis, and reduce amylopectin content. The obvious reduction of amylopectin contents would inevitably reduce total starch content (Table 1). Thus, our results showed that SGBP phosphorylation plays a critical role in starch biosynthesis.

**Fig. 8:**
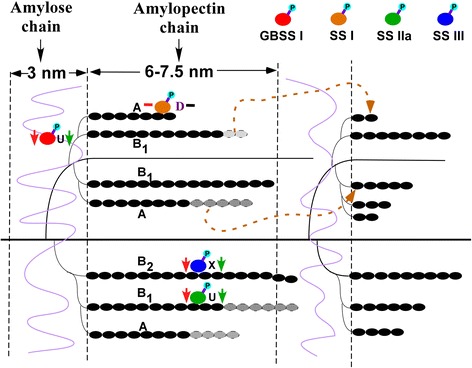
Schematic representation of amylose and amylopectin synthesis in the endosperm. Proteins with reduced levels under drought stress are indicated by the letter D. Proteins with increased levels under drought stress are indicated by the letter U. Proteins not detected in this study are indicated by the letter X. Down arrows (on the right with green colour) indicate that the phosphorylation levels were reduced under drought stress. The horizontal line indicates that the phosphorylation levels did not change under drought stress. Down arrows (on the left with red colour) indicate that the transcription level of these phosphoproteins were reduced under drought stress and the horizontal line (on the left with red colour) indicates that the transcription levels did not change under drought stress. Phosphoproteins are indicated by a lowercase letter p

Based on our results and previous reports, we propose putative pathways of amylose and amylopectin biosynthesis in response to water-deficit conditions (Fig. 8). The expression of starch biosynthesis-related proteins, such as GBSS I and SS II-a showed tiny increase, SSI was decreased significantly when subjected to the water-deficit treatment (Fig. 2). The phosphorylation levels of three key starch biosynthesis-related proteins were also reduced markedly by the water-deficit treatment as shown in Table 3, which could affect the activity of starch biosynthesis enzymes and their interaction, and result in reduced total starch content and grain yield. This also showed that the transcription levels of these genes are generally consistent with their phosphorylation levels but a little different with protein levels.

## Conclusions

Water-deficit treatment significantly reduced spike grain numbers and weight, total starch and amylopectin content, and grain yield. Proteome analyses found that the expression quantity of SS II-a was reduced markedly when subjected to water deficit stress. Phosphoproteome analysis of SGBPs found that the phosphorylation levels of the main starch synthesis enzymes such as GBSS I, SS II-a, and SS III were decreased significantly under water-deficit. Furthermore, several key genes related with starch biosynthesis such as *SS III*, *SBE II-b* and *AGPL I* were downregulated significantly at 15 DPA. Our results demonstrated that the decrease in transcription expression, protein expression and phosphorylation levels of starch biosynthesis-related enzymes under water-deficit condition are responsible for the reduction in total starch content and grain yield.

## Additional files

Additional file 1: Table S1. Efficiency and R^2^ values (coefficient of determination) of primer pairs. These values were determined using standard curves. **Table S2.** Granule-binding proteins identified by MALDI-TOF/TOF-MS. **Table S3.** Peptide information for the identified starch granule-associated proteins with at least twofold differences. **Table S4.** Total phosphorylated sites and phosphoproteins identified in this study. **Table S5.** Identification of proteins related to starch synthesis by matrix-assisted laser desorption ionization time-of-flight mass spectrometry (MALDI-TOF MS). (ZIP 124 kb)

Additional file 2: Figure S1. Experimental workflow used in this study. DPA: days post anthesis. 1, 2, and 3 represent the three biological replicates. **Figure S2.** Scanning electron microscopy images for evaluation of the purity of the starch granules from the Chinese wheat cultivar Jingdong 17. **Figure S3.** SDS-PAGE of proteins in supernatant after successive washing steps, one, two and three times, respectively (1–3). (ZIP 1339 kb)

## Abbreviations

ACN: Acetonitrile
AGPase: ADP-glucose pyrophosphorylase
AGPL I: Adenosine 5 diphosphate glucose pyrophosphorylase large subunit I;
AGPS II: Adenosine 5 diphosphate glucose pyrophosphorylase small subunit II
CID: Collision induced dissociation
DBE: Starch-debranching enzyme
DPA: Days post-anthesis
DTT: Dithiothreitol
GBSS I: Granule-bound starch synthase
ISA: Isoamylase
LC-MS/MS: Liquid chromatography coupled to tandem mass spectrometry
MALDI-TOF/TOF-MS: Matrix-assisted laser desorption/ionisation time-of-flight tandem mass spectrometry
NCBI: National Center for Biotechnology Information
P^3^DB: Plant protein phosphorylation data base
PUL: Pullulanase
qRT-PCR: Real-time quantitative reverse transcription polymerase chain reaction
SBE: Starch-branching enzyme
SEM: Scanning electronic microscope
SGBPs: Starch granule-binding proteins
SS: Starch synthase
TFA: Trifluoroacetic acid
