# Supplementary figures and images for: In vivo phosphoproteome characterization reveals key starch granule-binding phosphoproteins involved in wheat water-deficit response
Source: BMC Plant Biol. 2017 Oct 23;17:168. doi: 10.1186/s12870-017-1118-z (PMC5651632; doi:10.1186/s12870-017-1118-z)

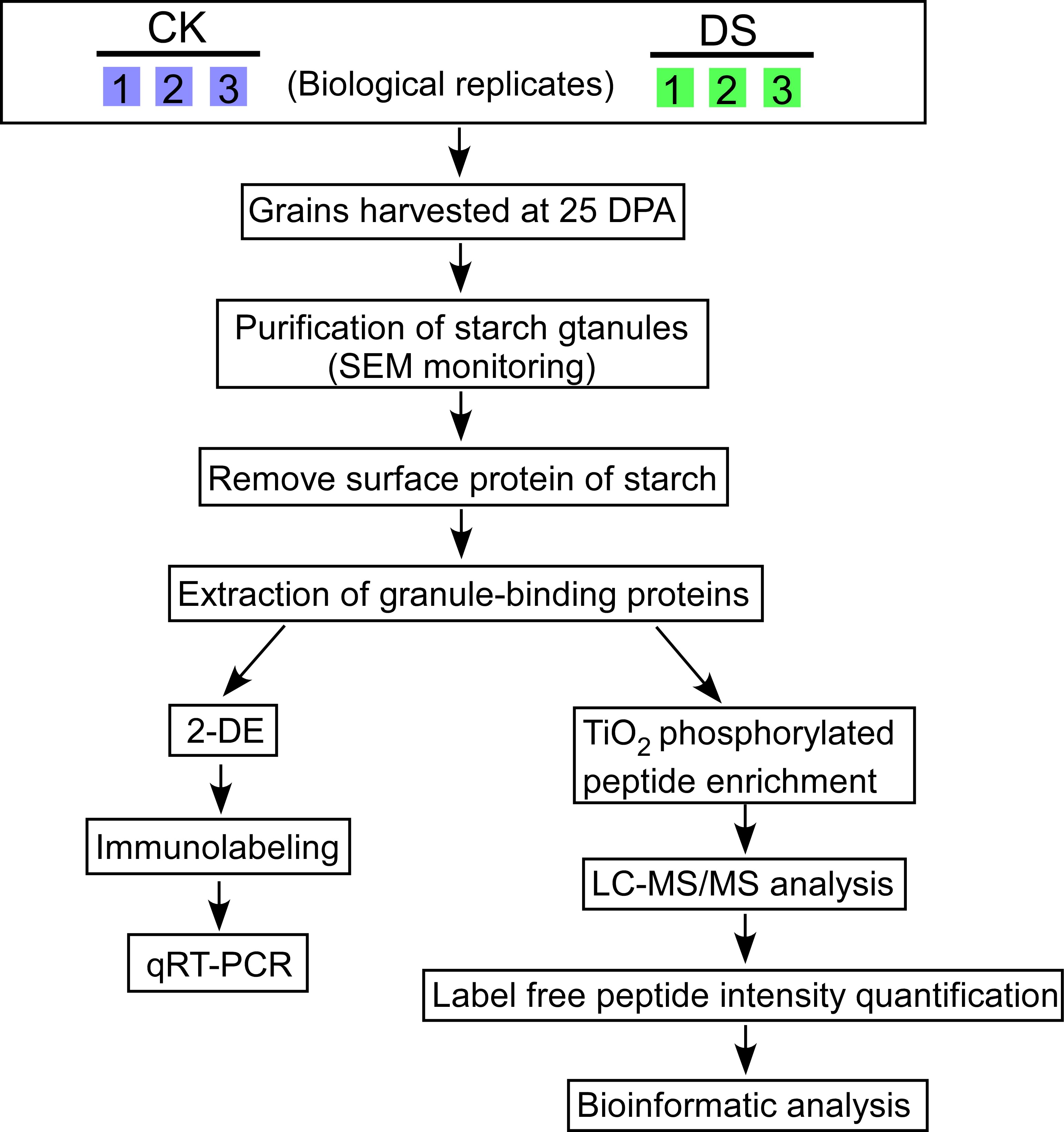

Supplement: Supplementary file 2 — Experimental workflow used in this study. DPA: days post anthesis. 1, 2, and 3 represent the three biological replicates. Figure S2. Scanning electron microscopy images for evaluation of the purity of the starch granules from the Chinese wheat cultivar Jingdong 17. Figure S3. SDS-PAGE of proteins in supernatant after successive washing steps, one, two and three times, respectively (1–3). (ZIP 1339 kb) [file 12870_2017_1118_MOESM2_ESM.zip › Additional File 2 Fig-S1.JPEG]

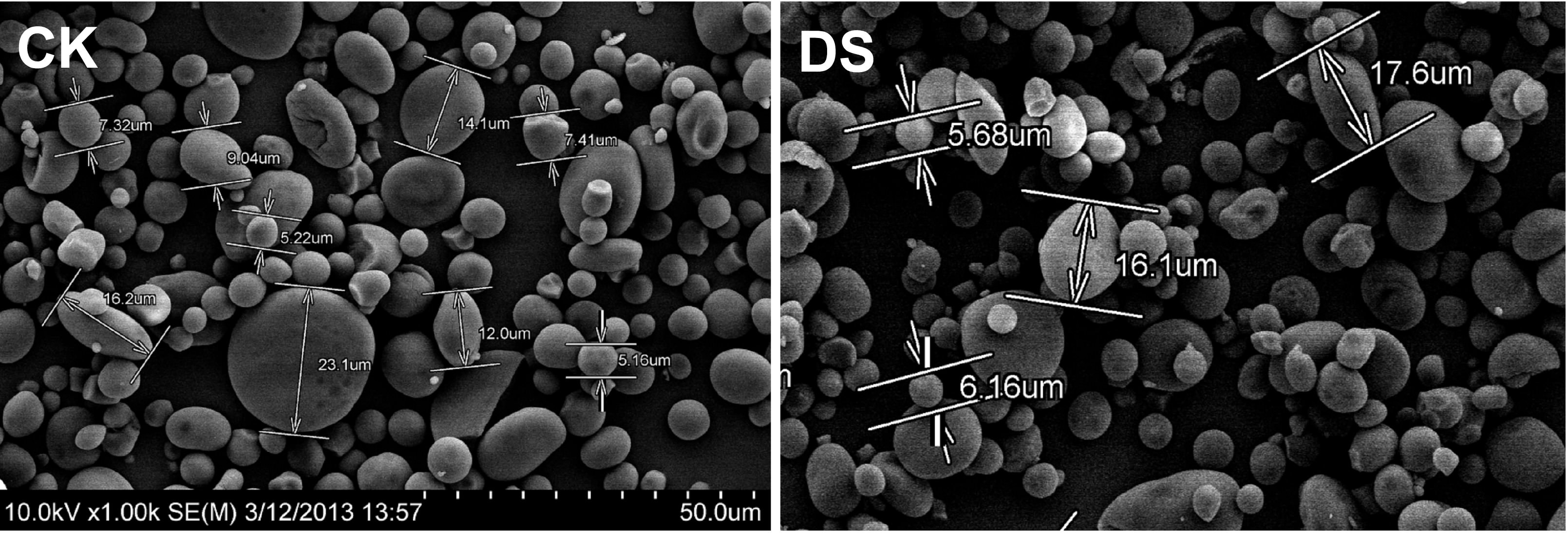

Supplement: Supplementary file 2 — Experimental workflow used in this study. DPA: days post anthesis. 1, 2, and 3 represent the three biological replicates. Figure S2. Scanning electron microscopy images for evaluation of the purity of the starch granules from the Chinese wheat cultivar Jingdong 17. Figure S3. SDS-PAGE of proteins in supernatant after successive washing steps, one, two and three times, respectively (1–3). (ZIP 1339 kb) [file 12870_2017_1118_MOESM2_ESM.zip › Additional File 2 Fig-S2.JPEG]

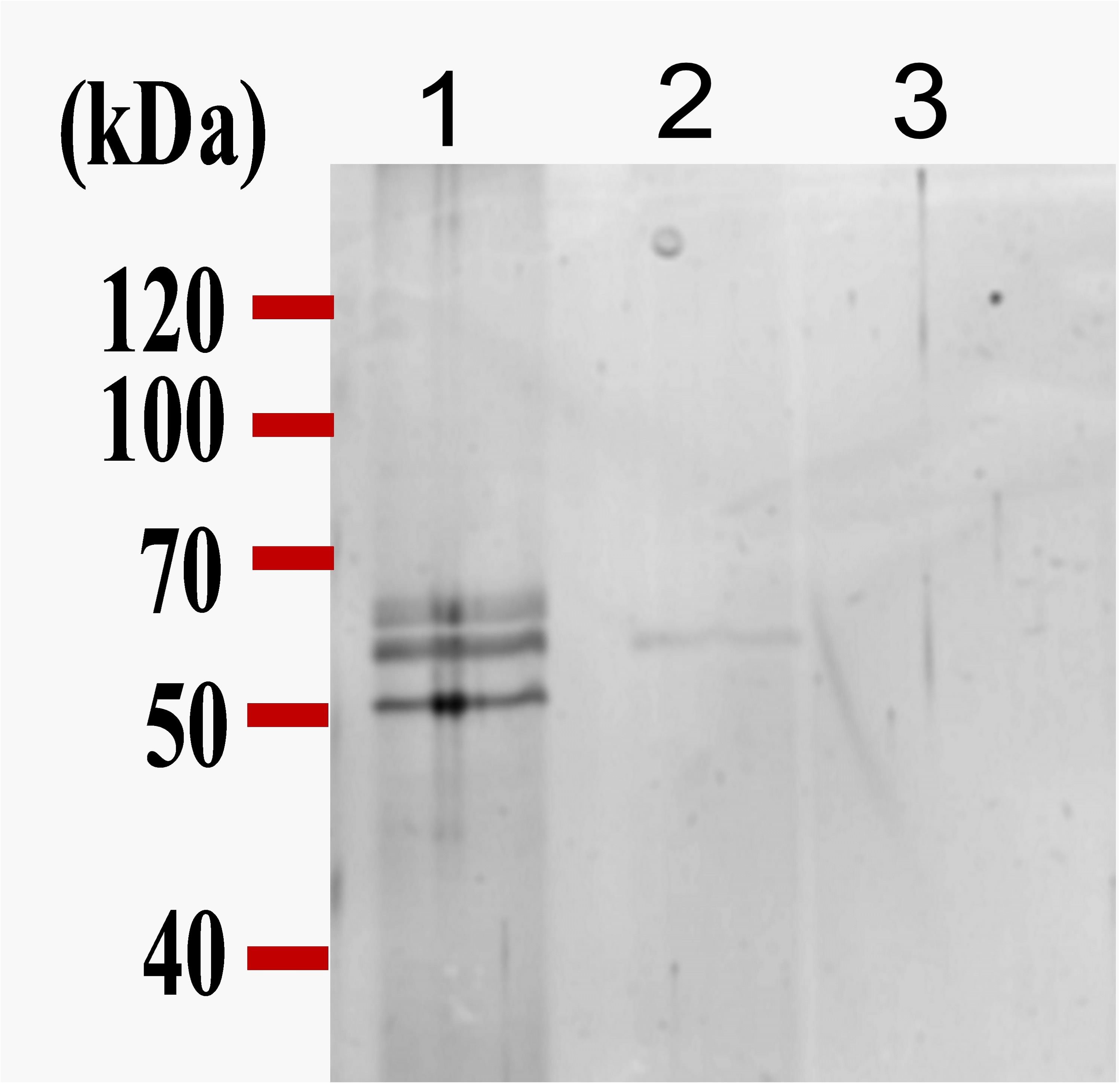

Supplement: Supplementary file 2 — Experimental workflow used in this study. DPA: days post anthesis. 1, 2, and 3 represent the three biological replicates. Figure S2. Scanning electron microscopy images for evaluation of the purity of the starch granules from the Chinese wheat cultivar Jingdong 17. Figure S3. SDS-PAGE of proteins in supernatant after successive washing steps, one, two and three times, respectively (1–3). (ZIP 1339 kb) [file 12870_2017_1118_MOESM2_ESM.zip › Additional File 2 Fig-S3.JPEG]
